# A Score of the Ability of a Three-Dimensional Protein Model to Retrieve Its Own Sequence as a Quantitative Measure of Its Quality and Appropriateness

**DOI:** 10.1371/journal.pone.0012483

**Published:** 2010-09-07

**Authors:** León P. Martínez-Castilla, Rogelio Rodríguez-Sotres

**Affiliations:** 1 Departamento de Bioquímica–Facultad de Química, Universidad Nacional Autónoma de México, Ciudad de México, Distrito Federal, Mexico; 2 Centro de Ciencias de la Complejidad, Universidad Nacional Autónoma de México, Ciudad de México, Distrito Federal, Mexico; University College Dublin, Ireland

## Abstract

**Background:**

Despite the remarkable progress of bioinformatics, how the primary structure of a protein leads to a three-dimensional fold, and in turn determines its function remains an elusive question. Alignments of sequences with known function can be used to identify proteins with the same or similar function with high success. However, identification of function-related and structure-related amino acid positions is only possible after a detailed study of every protein. Folding pattern diversity seems to be much narrower than sequence diversity, and the amino acid sequences of natural proteins have evolved under a selective pressure comprising structural and functional requirements acting in parallel.

**Principal Findings:**

The approach described in this work begins by generating a large number of amino acid sequences using ROSETTA [Dantas G *et al.* (2003) *J Mol Biol* 332:449–460], a program with notable robustness in the assignment of amino acids to a known three-dimensional structure. The resulting sequence-sets showed no conservation of amino acids at active sites, or protein-protein interfaces. Hidden Markov models built from the resulting sequence sets were used to search sequence databases. Surprisingly, the models retrieved from the database sequences belonged to proteins with the same or a very similar function. Given an appropriate cutoff, the rate of false positives was zero. According to our results, this protocol, here referred to as Rd.HMM, detects fine structural details on the folding patterns, that seem to be tightly linked to the fitness of a structural framework for a specific biological function.

**Conclusion:**

Because the sequence of the native protein used to create the Rd.HMM model was always amongst the top hits, the procedure is a reliable tool to score, very accurately, the quality and appropriateness of computer-modeled 3D-structures, without the need for spectroscopy data. However, Rd.HMM is very sensitive to the conformational features of the models' backbone.

## Introduction

Advances in molecular biology techniques and the development of both computer hardware and bioinformatic software have yielded an impressive amount of annotated protein sequences, but most, with unknown three-dimensional structures. How the primary structure of a protein leads to a three-dimensional fold, and in turn determines its function, remains an elusive question. Amongst other reasons, the huge number of possible primary sequences does not seem to lead to an equivalent diversity in folding patterns [1]. In addition, the same sequence may adopt two different folds as in the prion protein [2], while some sequences have a remarkable flexibility, as in Calmodulin [3]. All of these data indicate of a degree of informational degeneracy between the sequence and folding codes. On the other hand, the wealth of amino acid sequences available in current databases deriving from natural proteins does carry information shaped by a variety of selective pressures, including the structural requirements of the final three-dimensional-fold, the *in vivo* folding pathway, protein-protein functional interactions, meaningful structural transitions, and catalytic functions. All of these factors act together during evolution, and extensive research is required to identify roles for individual positions in the amino acid sequence of a particular protein. Notably, recent advances in enzyme design indicate a linkage between a folding pattern and its adequacy to host a particular active site geometry [4].

The reverse folding problem, *i.e.* going from the 3D-coordinates to the sequence, appears to be more attainable [5]. In addition, recent years have seen the upcoming of improved force fields to calculate non-bonded interactions in macromolecular three-dimensional structures, and better strategies to produce plausible three-dimensional structural models for sequences with unknown structure [6]–[9]. Amongst those programs, ROSETTA has achieved an outstanding success in the assignment of amino acids compatible to a 3D-fold defined only by its backbone [4], [10], [11]. We decided to take advantage of this property to generate a large number of amino acid sequences consistent with the 3D backbone of known proteins. This approach has been shown to enrich sequence-derived evolutionary models with structural information for various purposes [12]–[14]. However, we decided to completely exclude biological data from the assemblies, and work on a fixed backbone. In theory, the set of sequences generated in this way would be devoid of information related to the protein function, other than structural requirements specific to the particular 3D-structure selected. To integrate the information present in the huge number of sequences generated by ROSETTA, we used hidden Markov models, as implemented in HMMER [15] (http://hmmer.janelia.org). HMMER is well known for its robustness in the generation of reliable statistical models of protein sequence alignments. The results shown in this paper are surprising, because the hidden Markov models generated by ROSETTA design and HMMER (Rd.HMM) were able to recover, from the protein sequence databases, only those sequences with an strongly related, or identical function to the one of the original template. Thus, ROSETTA was able to imprint the sequence alignment with fine details of the folding patterns, and apparently many of these details are related to the the adequacy of the original 3D-fold to host a specific function. In addition, this scheme turned out to be appropriate for the assessment of modeled three-dimensional structures. The concept is similar to the one used by Lüthy et al [16], but our approach is not limited to the comparison with the modeled amino acid sequence only, but scores the structure against the entire sequence database and, notably, can be easily tuned to completely eliminate false positives. However, our approach is very sensitive to structural perturbations and it works well only if the backbone conformation of the model presents a distribution of bond lengths, angles and dihedrals similar to the one found in X-ray solved three-dimensional protein structures. This conformational state is here referred to as “crystal-like equilibrium conformation”.

## Results

### Conservation and pseudo-phylogenetic relationships in the alignment of the ROSETTA sequences

**Figure 1:**
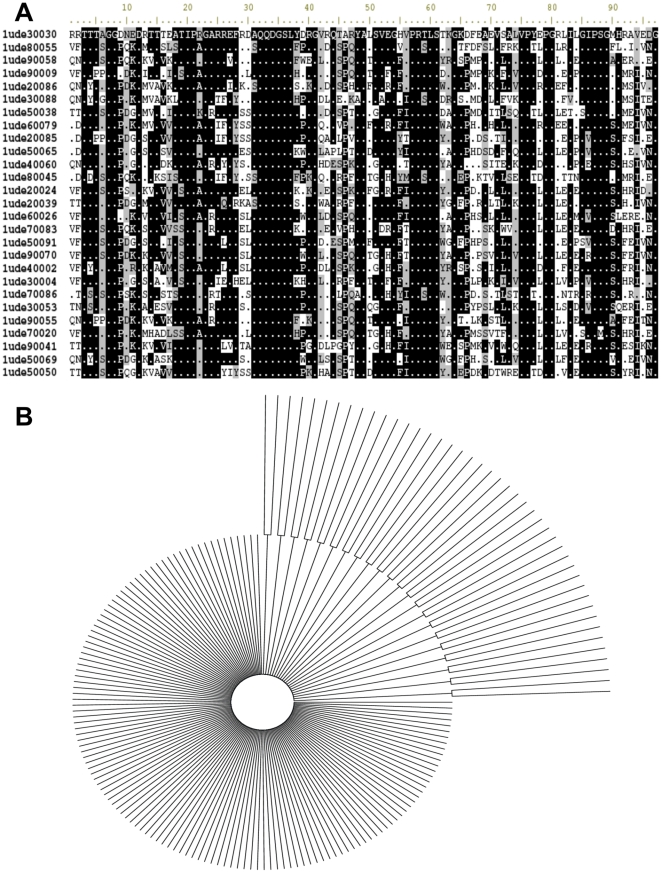
Sequence analysis of ROSETTA-rebuilt 3D structures for *Pyrococcus horikoshii* soluble inorganic pyrophosphatase (1UDE). A) Fragment of the alignment of 40 selected sequences from the Rosetta-design rebuilt sequences, for clarity, only positions from 4 to 96 and sequences from 1 to 27 are shown. B) A fragment of the majority-rule consensus bootstrap tree for the sequences generated by ROSETTA design, and partially shown in A (sequence names have been omitted).

**Figure 2 pone-0012483-g002:**
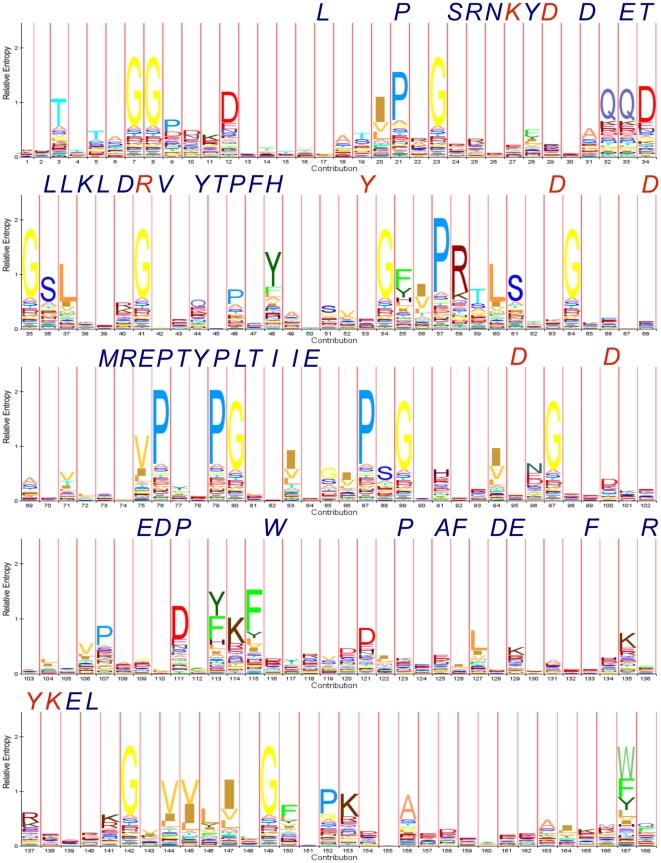
HMM logo of the Rd.HMM for the 1UDE inorganic pyrophosphatase. This model was produced with the whole set of sequences of 800 ROSETTA-rebuilt 3D structures for *Pyrococcus horikoshii* soluble inorganic pyrophosphatase, the figure was generated with the web service HMM logos [17].

The obvious first step is the analysis of the resulting alignments of the functionally unconstrained ROSETTA generated sequences. Figure 1 shows a subset of an alignment of 800 sequences (panel A) generated by ROSETTA from the three-dimensional fold of the *Pyrococcus horikishii* inorganic pyrophoshpatase (PDB entry 1UDE). The HMM logo [17] of the alignment (Fig. 2) reveals highly variable zones and a few invariant positions. In addition, the informational content is distributed unevenly along the sequence. The native amino acid corresponding to positions of subunit-subunit contacts (blue) or active site residues (red) are indicated in italics, above its logo column. Clearly, there is an almost complete loss of functional information in the artificial 3D-structures generated by ROSETTA design. This was expected since ROSETTA design lacks the function-related information required to establish such conservation. However, the program should preserve the information related to the stability of the folding pattern. Furthermore, amino acid assignment by ROSETTA design depends on Monte Carlo searches, so each new amino acid sequence assigned by this program should be independent of the previous one. For this reason, we expected the ROSETTA-generated sequences to have very little pseudo-phylogenetic structure, and we analyzed these 800 sequences with PhyML [18], using the JTT+i+g+f substitution model, as recommended by the program ProtTest [19]. In the consensus bootstrap phylogenetic tree (partially shown in Figure 1B) , the phylogenetic structure is almost absent and the support values were very low (the highest support value was 82, and the lowest one was 0, with an average of 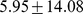 and mode 0, comprising nearly 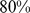 of the nodes). Therefore, sequence assignment by ROSETTA design is evolutionarily uncommitted, and the sequence conservation we observed in the alignments must derive from the structural and chemical constraints imposed by the backbone coordinates fed into to the program. As a consequence, functional information would be lost during the ROSETTA design step, with the exception of those positions where the structural and the functional requirements coincide.

Another observation on the alignment in Figures 1A and 2 is the remarkable tendency of invariant positions to contain Gly, Pro and Asn. These amino acid residues are known to adopt 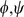 angles departing from the generally allowed values [20] and, as a consequence, the ROSETTA rotamer database does not contain many alternative choices for strained positions. In fact, an analysis of the structural features of these invariant positions confirmed the occurrence of strained dihedrals (this is shown below under “Rd.HMM of highly mobile proteins”). However and as expected, the same amino acids may appear in highly variable positions, therefore, the presence of a Gly, Pro or Asn in the amino acid sequence gives little information by itself, a well known fact.

### Consideration on the use of extended rotamer libraries in ROSETTA

ROSETTA design can be made to consider extended rotamer libraries with the use of the -exn (n = 1,2, 3 and 4), and the -extrachi_cutoff flags. The use of this flags resulted in a small increase in the number of hits in the HMMer list and an small increase in the score, yet computation time and the use of computer memory scaled up significantly. For instance a 1010 amino acid protein would require more than 64 Gbytes of memory with the extra-chi cutoff set to 1 and consumed 36 GBytes if only -ex1 and -ex2 flags are set. Running ROSETTA in parallel would require an amount of memory proportional to the number of computer processors requested. An additional consideration is computation time. The whole process may take from 6 to 12 hrs for small proteins (using one 3 GHz 64-bit processor), but it may take nearly a week for a 1000 amino acid-long protein chain. Therefore, the use of the -ex3, -ex4 or -extrachi_cutoff flags is not recommended, except for small proteins.

### Database search with hidden Markov models

**Figure 3:**
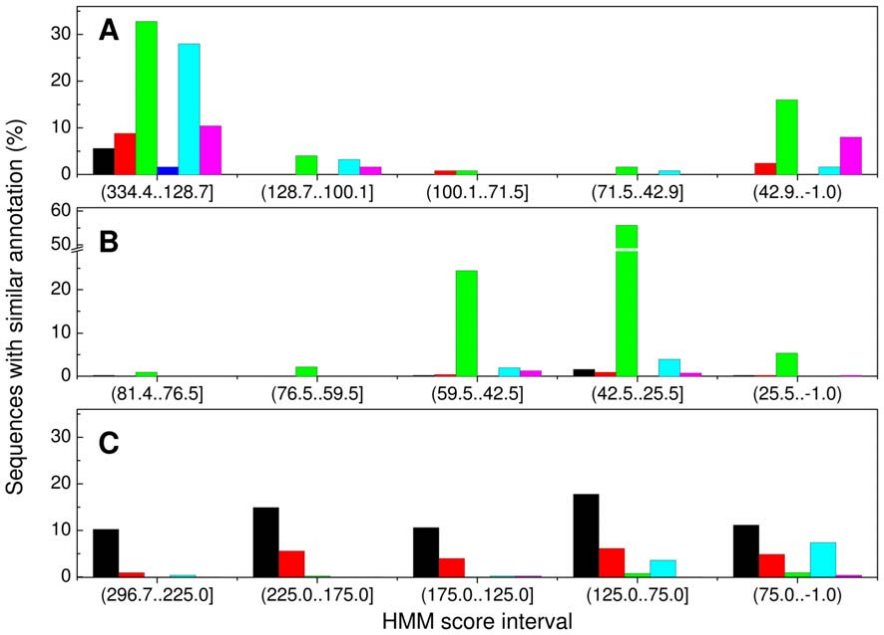
Classification of database annotations in the list from Rd.HMM searches correponding to the soluble inorganic pyrophosphatases. The Rd.HMM were generated for the pyrophosphatases from *Saccharomyces cerevisiae* (1E9G; panel A), *Pyrococcus horikoshii* (1UDE; panel B), and *Streptococcus gordonii* (1K20; panel C). The search results lists were classified in bins according to the HMM score and each bin was subdivided by keywords in a mutually exclusive fashion. For panel A and B the black bars correspond to hits annotated as PDB structure of a pyrophosphatase, red bars include hits annotated as putative or predicted pyrophosphatase, green bars correspond to sequences annotated as pyrophosphatase, blue bars include the nucleosome remodeling factor (that has pyrophosphatase activity). In panel C, black bars are PDB entries of manganese-dependent pyrophosphatases, red bars are sequences annotated simply as pyrophosphatases, and green bars correspond to sequences known to share a domain with manganese-dependent pyrophosphatases, such as DRTGG or DHH domain-containing proteins, TrkA phosphoesterase, or exopolyphosphatase prune. In all three panels, cyan bars correspond to predicted, hypothetical or putative proteins, and magenta bars include everything else. Regardless of the annotation, the hits in these last two categories (cyan and magenta bars) and in the two highest Rd.HMM score intervals were manually searched in the NCBI databases in “genpept” format, and all were found to share at least a domain of the same class as the starting structure.

The sequences from the ROSETTA design models were aligned and used to build a hidden Markov model using HMMER, the resulting ROSETTA-HMMER model (Rd.HMM for brevity) was calibrated and used to search the NCBI-nr database using the default threshold (E-value smaller than or equal to 10). Sets of ROSETTA-designed sequences were generated starting with the three-dimensional structure of the pyrophosphatase from Yeast (family one Eukaryotic type; pdb 1E9G) and with the one from family two manganese-dependent pyrophosphatase (1K20), and were used to build two additional Rd.HMM (Fig. 1, and Table S1). Surprisingly, amongst the recovered sequences for the Rd.HMM, those containing annotations were soluble inorganic pyrophosphatases. The Rd.HMM obtained for the yeast enzyme recovered family I pyrophosphatases of the Eukaryotic type (Fig. 3A, and Table S1), while the Rd.HMM from 1UDE recovered sequences mostly of the bacterial type (Fig. 3B, and Table S1). The recovered sequences were grouped according the their Rd.HMM score and within each group the annotations were scanned for keywords indicative of their nature. Where the database heading annotation was vague, we reviewed the corresponding gi entries in “genpept” format at the NCBI's site, specially for the sequences with high scores. In every case analyzed, we found a putative soluble inorganic pyrophoshatase domain in the corresponding sequence. Table S1 shows the whole list of sequences recovered, including their respective Rd.HMM score, their E-values, and in the case of 1UDE the sequence alignment; this table also presents their biological source and the annotation. In the Rd.HMM from the bacterial family-I pyrophosphatase, the starting crystal was hit number 3, and had a Rd.HMM score very close to the top one and highly significant E-value (Table S1). The first sequence found from a non-Archaeon was hit number 14 (belonging to a cyanobacteria), and the Rd.HMM score was significantly reduced, and Eukarya representatives were only plants and a cilliate. This may not be surprising, since plants have been found to possess inorganic soluble pyrophosphatases of both the bacterial and fungal type, while animals seem to have proteins of the fungal type only [21], but it clearly shows the ability of this strategy to give a higher score to sequences that are more closely related to the amino acid sequence of the starting 3D-structure. In the case of the Mn-dependent inorganic pyrophosphatase (1K20; Fig. 3C, and Table S1), some hits belong to the DHH family and the TrkA domain-containing proteins, which are known to have a inorganic pyrophosphatase/exopolyphosphatase domain. In addition, the sequence of the protein used to produce the hidden Markov model was invariably found amongst the top hits (see Table S1). In addition, Table S1 includes the search-results from two Rd.HMM corresponding to the N-terminal domain of the *Bacillus subtilis* Mn-dependent inorganic pyrophosphatase. The first one was prepared as mentioned before, and the second one includes the natural sequence in the alignment used to build the hidden Markov model. The effect of including this last additional sequence was a substantial increase in the Rd.HMM scores (roughly 6 times higher). This last result and the logo in Figure 2 indicate a loss of the the function-related information in the sequences generated by ROSETTA design.

While the above results look promising, the soluble inorganic pyrophoshatases constitute a family with many members and have a unique distorted barrel folding, so we decided to build a similar Rd.HMM starting from a protein structure with a more widespread folding pattern. The TIM-like 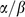 barrels are present in many diverse proteins and enzymes, either isolated or as a domain of multidomain proteins. These proteins present several activities and functions other than the triose phosphate isomerase catalytic activity. We therefore selected the triose phsophate isomerase from five different organisms, because there are several different structures solved from species with distant phylogenetic relationships. To further test the power of this Rd.HMM models to recover sequences from the database, we built individual models for each protein, and a combined model including the whole set (3000 sequences). To produce a structurally meaningful alignment the five original sequences were first aligned using TOPOFIT [22], and the gapping pattern from each sequence was propagated to its ROSETTA-designed descendants, as described in the methods section. The natural sequences were then eliminated and the resulting alignment of all the ROSETTA-designed variants was used to build the hidden Markov model, with HMMER.

As before, the resulting searches produced a list of hits containing almost exclusively triose phosphate isomerases, or unannotated sequences. Sets from each individual protein Rd.HMM were subsets of the set recovered with the combined full model, this last Rd.HMM recovered 418 unique entries from the SWISS-PROT database. Only 3 sequences recovered by both Rd.HMM for Yeast (PDB entry 1NEY) and the the one for *Caenorhabditis elegans* (PDB entry 1MO0) proteins were not included in the full Rd.HMM list (Table 1). These three hits had negative Rd.HMM scores, E-values of negligible statistical significance (above 1), and were not annotated as triose phosphate isomerases (TPI). On the other hand, within the hits of the combined Rd.HMM , most sequences were annotated as TPI, putative TPI, or unknown. When further analyzed, the unknown proteins turned out to be possible TPI, but poorly characterized, or bifunctional phosphoglycerate mutase
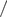
TPI proteins (see Table 1, hit 250 from full Rd.HMM, as an example). In these last cases, the HMMER alignment revealed matches to only the TIM-barrel domain, despite the similarity in the 3D folding patterns of both enzyme domains (see the column “N. dom.” in Table S2, spreadsheet named “full”, row 252, which indicates that only one domain gave a significant score). All of the Rd.HMM for individual crystals did recover a slightly smaller set of sequences (Table 2). Each individual Rd.HMM did recover the natural sequence from the starting PDB-structure with the highest score and the smallest E-value. The sequences recovered by the combined Rd.HMM and missing on the search-outputs of at least one individual Rd.HMM were less than 3% of the recovered sequences. From these, the hits with the highest score for each individual Rd.HMM are presented in Table 2. Only one of those hits showed a positive Rd.HMM score (including those not shown in Table 2), but even so, all were annotated as TPI. In accordance with the tendency of the Rd.HMM to give better scores to those sequences with closer phylogenetic relationships, the low score hits in Tables 1 and 2 correspond to sequences from bacterial sources, while the crystals chosen to generate the full Rd.HMM were all from proteins belonging to Eukaryotes (for the full data sets used in the preparation of Tables 1 and 2 see Table S2).

**Table 1:** Selected hits from the Rd.HMM search starting with the combined model of 6 crystals (6-combined), and for each individual crystal (PDB entries 1KV5, 1R2R, 1NEY, 1TPH, 1M6J, 1MO0).

Starting Crystal	Hit No.	NCBI-nr gi	PDB entry	Score^a^	E-value^a^	Biological Source	DB annotation^b^
6-combined	1	136062		248.7	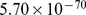	*Macaca mulatta*	TPI
6-combined	2	117935064		248.2	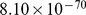	*Rattus norvegicus*	TPI 1
6-combined	3	136066	1R2R	244.8	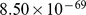	*Oryctolagus cuniculus* (Rabbit)	0801190A TPI
6-combined	17	45382061	1TPH	228.3	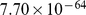	*Gallus gallus*	TPI 1
6-combined	24	17536593	1MO0	223.7	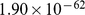	*Caenorhabditis elegans*	TPI family member (tpi-1)
6-combined	39	6320255	1NEY	209.7	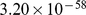	*Saccharomyces cerevisiae*	TPI
6-combined	43	1730005	1CI1	206.1	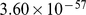	*Trypanosoma cruzi*	TPI
6-combined	48	730975	1KV5, 4TIM	197.7	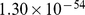	*Trypanosoma brucei*	TPI
6-combined	51	1351275	1AMK	194.4	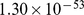	*Leishmania mexicana*	TPI
6-combined	54	28380171	1M6J	187.7	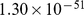	*Entamoeba histolytica*	Structure of TPI from *E. histolytica*
6-combined	75	110833186		127.2	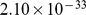	*Alcanivorax borkumensis* SK2	TPI
6-combined	100	1730000		118.4	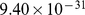	*Heliothis virescens* (Tobacco budworm moth)	TPI
6-combined	125	153932906		109.9	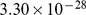	*Clostridium botulinum* A str. ATCC 19397	TPI
6-combined	150	77359822		106	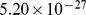	*Pseudoalteromonas haloplanktis* TAC125	TPI
6-combined	175	167625063		101	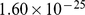	*Shewanella halifaxensis* HAW-EB4	TPI
6-combined	200	84624726		96.7	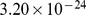	*Xanthomonas oryzae* pv. oryzae MAFF 311018	TPI
6-combined	250	15643452		90.5	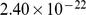	*Thermotoga maritima* MSB8	phosphoglycerate kinase/TPI
6-combined	300	162147716		80.6	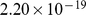	*Gluconacetobacter diazotrophicus* PAl 5	putative TPI
6-combined	350	23500597		63.2	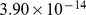	*Brucella suis* 1330	TPI
6-combined	400	148284061		29.6	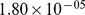	*Orientia tsutsugamushi* str. Boryong	TPI
6-combined	416	15644823		−39.5	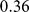	*Helicobacter pylori* 26695	TPI
6-combined	417	28493269		−41	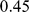	*Tropheryma whipplei* str. Twist	TPI
6-combined	418	28572619		−41.7	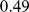	*Tropheryma whipplei* TW08/27	TPI
1KV5	1	730975	1KV5, 4TIM	208.3	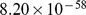	*Trypanosoma brucei*	TPI
1R2R	1	136066	1R2R	221	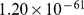	*Oryctolagus cuniculus* (Rabbit)	0801190A TPI
1NEY	1	6320255	1NEY	190.2	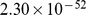	*Saccharomyces cerevisiae*	TPI
1NEY	413	149246972		−28.3	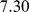	*Lodderomyces elongisporus* NRRL YB-4239	conserved hypothetical protein
1TPH	1	45382061	1TPH	208.7	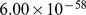	Gallus gallus	TPI 1
1M6J	1	28380171	1M6J	198	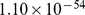	*Entamoeba histolytica*	Structure of TPI from *E. histolytica*
1MO0	1	17536593	1MO0	172.1	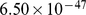	*Caenorhabditis elegans*	TPI family member (tpi-1)
1MO0	409	32035699		−26.1	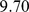	*Actinobacillus pleuropneumoniae* serovar 1 str. 4074	COG2931: RTX toxins and related Ca2+-binding proteins
1MO0	411	15609294		−26.3	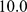	*Mycobacterium tuberculosis* H37Rv	UDP-mgpAAL, MurF

Rd.HMM search sets obtained with each individual PDB file were a subset of the 6-combined Rd.HMM search, but the order changed and as shown below each recovered its own sequence on top of the list. The table includes the 3 hits found by individual Rd.HMM and absent from the 6-combined (E-values>1). Notes:

a For the corresponding Rd.HMM.

b TPI = triose phosphate isomerase; UDP-mgpAAL = UDP-N-acetylmuramoylalanyl-D-glutamyl-2,6-diaminopimelate- D-alanyl-D-alanyl ligase.

**Table 2:** Comparative analysis of the sets from the individual Rd.HMM results *vs.* the 6-combined Rd.HMM (see Table 1).

Starting Crystal	Total hits	Hits missing^a^	Top missing hit^b^				
			Hit number	Score^c^	E-value^c^	Biological Source	DB annotation
1KV5	413	6	413	−23	0.034	*Helicobacter pylori* J99	TPI
1R2R	417	2	417	−41	0.450	*Tropheryma whipplei* str. Twist	TPI
1NEY	414	6	411	−6.3	0.0031	*Campylobacter curvus* 525.92	TPI
1TPH	417	2	417	−41	0.450	*Tropheryma whipplei* str. Twist	TPI
1M6J	407	12	411	−6.3	0.0031	*Campylobacter curvus* 525.92	TPI
1MO0	411	10	406	3.6	0.00075	*Campylobacter jejuni* subsp. doylei 269.97	TPI

Notes:

a Each sequence DB entry found in the 6-combined model (see Table 1) and absent from an individual results set is considered a missing hit.

b The missing hit with the highest score found is included, most have negative score and appear near the end of the list, yet they all were annotated as triose phosphate isomerases (TPI) from bacterial sources.

c For the corresponding Rd.HMM.

Models built from other TIM barrels like the phosphoribosylanthranilate isomerase and the 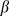 1,4-endoglucanase were equally selective, producing database subsets of proteins reported to have the same activity, with the exception of some annotated as “unknown”, “conserved”, or “putative” proteins. In all cases, whenever we looked for the sequence details at NCBI's site, the sequences turned out to be the same type of protein as the one used to produce the model (see Figure S1).

### Hidden Markov Models of unusual proteins

Because the pyrophosphatases and the TIM barrels mentioned above are widely distributed, we decided to test the stringency of the method with rare proteins. TOP7 (PDB entry 1QYS) is an artificial fold whose design was ROSETTA-assisted. On the other hand, the putidaredoxin (PDB entry 1XLQ) is an unique electron carrier from bacteria, bearing a relationship with ferredoxins. The 2Fe-2S ferredoxin superfamily possess a rare fold because it has only short segments of repetitive secondary structure (see d1xlqa1 entry in the SCOP [23] database). Table 3 shows selected hits recovered from the Rd.HMM search with both of the above structures. In the two cases the Rd.HMM retrieved their native sequence, but Top7 retrieved only itself, while 1XLQ retrieved ferredoxins, and the upper 7 hits belong to the putidaredoxin from *Pseudomonas putida* (native or after site-directed mutagenesis). We can then safely conclude that the Rd.HMM from X-ray resolved protein structures are highly selective, and the number of relatives present in the sequence database searched did not affect this selectivity.

**Table 3:** Selected hits from the Rd.HMM of the three-dimensional structures for Top 7 (PDB entry 1QYS), the putidaredoxin (PDB entry 1XQL), and three *de novo* designed enzymes (PDB entries 3B5L, 3HOJ, and 2RKX).

Starting structure	Hit number	gi	PDB or RefSeq entry	Score^a^	Log (E-value^a^)	Biological Source	full description
1QYS	1	39654745	1QYS	52.7	−9.13	*de novo* design	Top7
1XQL	1 to 7	157831233^b^	1GPX , 1OQQ , 1PDX , 1OQR , 1PUT , 1R7S^c^	77.5 to 73.9	−16.49 to −15.38	*Pseudomonas putida*	Putidaredoxin (Fe 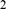 S 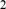 Ferredoxin)
1XQL	8	114327663	YP_744820.1	63.9	−12.39	*Granulibacter bethesdensis*	ferredoxin, 2Fe-2s
1XQL	20	27376160	NP_767689.1	51.1	−8.52	*Bradyrhizobium japonicum*	ferrodoxin
1XQL	50	83942806	ZP_00955267.1	41.2	−5.57	*Sulfitobacter sp*.	Fe-S cluster-binding protein
1XQL	100	14010742	NP_114221.1	36.7	−4.2	*Acinetobacter sp*.	ferredoxin
1XQL	150	116670546	YP_831479.1	31.6	−2.68	*Arthrobacter sp*.	ferredoxin
1XQL	200	157964264	YP_001499088.1	26.5	−1.14	*Rickettsia massiliae*	ferredoxin
1XQL	250	15604072	NP_220587.1	20	0.68	*Rickettsia prowazekii*	adrenodoxin
1XQL	272	42520681	NP_966596.1	17.1	1	*Wolbachia sp*.	ferredoxin, Fe-S cluster assembly system
1B5L	1	166007309	3B5L	202.7	−54.17	*de novo* design	novel engineered Retroaldolase: Ra-61
1B5L	3	33357315	1M4W	194.2	−51.62	*Nonomuraea flexuosa*	Thermophilic B-1,4-Xylanase
1B5L	373	157930095		−10.9	0.94	*Neocallimastix patriciarum*	endo-1,4-beta-xylanase
3HOJ	1, 2	21730711, 15897782	1LBF, NP_342387.1	214.8	−57.82	*Sulfolobus solfataricus* P2	IGPS^d^ with I3GP at 2.0 A resolution
3HOJ	3	166007310, 256599796	3B5V, 3HOJ^d^	210	−56.36	*de novo* design	novel engineered retroaldolase: RA-22
3HOJ	997	50415562	XP_457477.1	−14.9	0.89	*Debaryomyces hansenii* CBS767	hypothetical protein DEHA0B12276g (GATase1/IGPS^e^)
2RKX	1	170292384	2RKX	276	−76.24	*de novo* design	Cyclase subunit of ImGEPS^f^
2RKX	2	20149859	1GPW	275.8	−76.17	*Thermotoga maritima*	ImGPS^g^ Bienzyme Complex
2RKX	1268	153808218	ZP_01960886.1	1	0	*Bacteroides caccae* ATCC 43185	hypothetical protein BACCAC_02506. ProFARim^h^

Notes:

a For the corresponding Rd.HMM.

b gi number for 1GPX.

c All entries are the same protein as 1XLQ, but some present a few site-directed mutations.

d Entry 3HOJ replaced 3B5V.

e Indole-3-glycerol-phosphate synthase.

f Imidazoleglycerol_evolvedcerolphosphate synthase.

g Imidazole glycerol phosphate synthase.

h Phosphoribosylformimino-5-aminoimidazole carboxamide ribonucleotide isomerase.

The 3D-folds tested in Figure 1 to [Fig pone-0012483-g002] 3 and Tables 1 and 2 produced very selective Rd.HMM and the resulting sequence sets included proteins with the same or very similar biological activity. Although these data include several 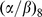 barrels, when analyzed in close detail, substantial changes can be found in the length and structure of several loops, the length of 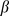-strands and 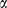-helices, and the distance between these various secondary-structure elements. However, conserved residues at the active site and other function-related information would be lost on the ROSETTA-designed sequences, yet, all of the sequences retrieved from the database belonged to proteins with the same or very similar function to the one used to produced the model. Therefore, the 3D-fold of these proteins appear to be finely tuned to host one biological function, when fine details of the 3D-structure are considered. To further test this proposal, we took advantage of the recent advances in *de novo* design of enzymes published by the group of David Baker [4], [11]. Rd.HMM were built with the coordinates of two retroaldolases [4] designed with two different 3D-templates (PDB entries 1B5L and 3HOJ), and the imidazoleglycerol-evolvedcerolphosphate synthase (PDB entry 2RKX) [11]. Table 3 includes the top hits of the resulting Rd.HMM search, where the sequence corresponding to the starting crystal from the database is in the top places of the list, with high score and low E-value. Interestingly, the other sequences in each search belong to the natural protein used as the template in the enzyme design, and its relatives. Table 3 includes one of the lowest hits of the list, where the sequence annotation in the database was clear. According to these data, the Rd.HMM was unable to discriminate sequences with completely different enzymatic activity, but with very similar 3D-fold. Clearly, the Rd.HMM encodes mostly, if not exclusively, the structural information of the starting 3D-coordinates.

**Figure 4:**
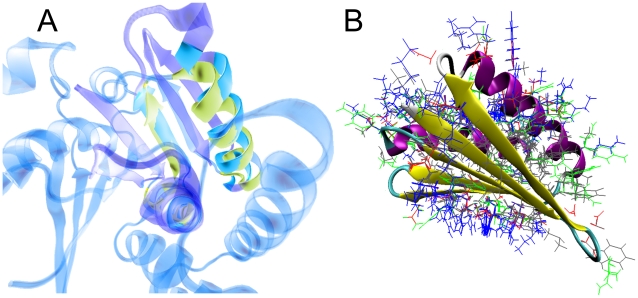
Evidence of the relationship between the Rd.HMM protocol and the three-dimensional structure of the protein. A) Cartoon representation of the TOPOFIT superposition of the TOP7 (1QYS) and the Bacterial glucose-inhibited protein (1XDZ). Cartoons are transparent, except where superposition was maximal. Only some sections of the backbone 3D structure were coincident, and the topology is also different, yet the Rd.HMM of 1QYS retrieved the sequence of one 1XDZ relative (refseq ZP_01740367.1) with a score of 13.9 (E-value 350). B) Superimposed ROSETTA-designed 3D-structures starting with the TOP7 protein (1QYS). The Rd.HMM found only the sequence of TOP7 (gi 39654745, 1QYS—A; Rd.HMM score 52.7, E-value 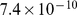.)

Considering the above result, we decided to extend some of the searches made with unusual proteins to find a sequence where structural information was available, even if the score was poor and the E-value lacked statistical significance. In the search results set from TOP7, we found a glucose-inhibited bacterial protein with unknown function (gi 126724524, ref ZP_01740367.1). The 3D-structure for this last sequence itself has not been determined, but it has high sequence similarity to the crystal corresponding to the PDB entry 1XDZ. TOP7 was found to have no sequence similarity to 1XDZ (blast2seq found no significant similarity), and their topologies were found to be different, yet TOPOFIT structural alignment revealed a region in 1XDZ with a coinciding spatial arrangement of secondary structural elements (Fig. 4A).

### How is Rd.HMM able to encode the traits of a three-dimensional structure in a Markov model?

Although we can not give a complete answer to the above question, to provide a visual idea of how the Rd.HMM can discriminate if one amino acid sequence is compatible with a certain 3D-fold, we created a superimposed view of 25 TOP7-derived ROSETTA-designed PDB files (Fig. 4B) From this image, ROSETTA design appears to be scanning the conformational space available at each position, the physicochemical and steric properties of compatible nearest neighbors, and the backbone conformational constraints. Similar images from other structures produced equivalent results.

As mentioned above, strained positions tend to be invariant, and the preceding and following positions tend also to show reduced variability. The distribution of such invariant positions and the spacing between them appears mostly conserved in the natural sequences contained in the Rd.HMM search results. In fact, these data could be used to create structurally aware alignments of the sequences, using structural information for only one or a few members of the protein family (see Figure S2).

In addition, although the hidden Markov models are unsuited to encode mutual information and long-range dependencies, if the structural features of one position reduce the possible choices of amino acids at neighbouring positions, this will impact the probability distribution of individual states (*i.e.* aa positions) in the corresponding hidden states of the Markov model built by HMMER. As a consequence, the Rd.HMM imply informational degeneracy because the Markov model may emit sequences that fail to fit into the original 3D-fold, along with a few that fit. In fact, this was found to be the case. With a given Rd.HMM most of the sequences emitted by the HMMER emit-module gave energy scores as poor as random sequences, when forced back into the original 3D backbone with ROSETTA design (not shown).

As a conclusion from the preceding observations, the Monte Carlo search made by ROSETTA design yields a set of sequences, which constitutes a robust signature of the 3D-fold provided. Subsequently, these sequences can be melded by HMMER into a hidden Markov model to provide a robust tool in the identification of natural sequences bearing a closely related native 3D-structure.

This last proposal is in agreement with all of the above considerations, and with the data discussed in the preceding section (Table 3 and Fig. 4).

### Rd.HMM of highly mobile proteins

**Figure 5:**
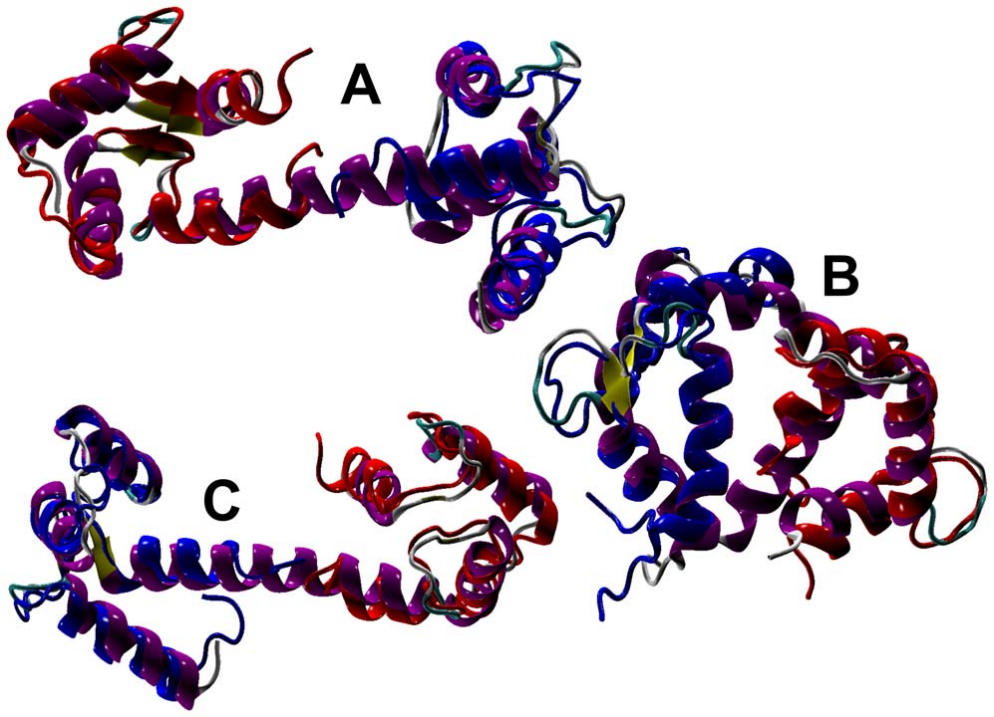
Cartoon representation of the Calmodulin NMR conformers whose Rd.HMM were able to retrieve sequences (see **Table 4**). The N-terminal (red, amino acids 1 to 79) and C-terminal (blue; amino acids 80 to 148) were superimposed independently to the corresponding region of the structure of Calmodulins in the PDB entry retrieved by each Rd.HMM. A) Superposition of 1CFF model 2 to 2F2O. B) Superposition of 1CFF model 8 to 2VRK. C) Superposition of 1CFF model 18 to 2GGZ.

Since we expected the Rd.HMM to be dependent on the protein structure, rather than on its sequence alone, we chose a protein with a highly mobile structure to analyze how the Rd.HMM from different conformers behaved. We selected a set of 26 conformers of Calmodulin derived from NMR-experiments (PDB entry 1CFF) and each one of the 26 models was submitted to the Rd.HMM protocol to generate a set of 150 variants per model. The sets from each individual conformer, and the resulting 3900 sequences from a joined set were used to generate the corresponding Rd.HMM. The search with such Rd.HMM produced an empty list, in most of the cases. However, some individual conformers produced Rd.HMM able to recover sequences from Calmodulins, and other proteins containing Calcium-binding EF-hands. Finally, when the crystal structure of the Calmodulin in the closed conformation (PDB entry 2HQW) was used to make a Rd.HMM, the search retrieved as many a 2821 calmodulins and calcium-binding proteins with EF-hands. Similar results were obtained with the crystal structures of Calmodulin from *Paramecium tetraurelia* (PDB entry 1CLM) and potato (PDB entry 1RFJ). A selected sample of this data is shown in Table 4. From these results, a clear relationship between the 3D-structure and the Rd.HMM emerges, because several conformers were unable to recover sequences from the database, but those who did, retrieved proteins belonging to the Calmodulin superfamily. In addition, in the three Rd.HMM from individual conformers where the search retrived some sequences, the list included a reference to a PDB entry, in this cases, the N-terminal and C-terminal domains of the NMR-conformer and the corresponding crystal structures were highly coincident (Fig. 5). Thus, these particular NMR-conformers adopted a 3D-structure matching the crystal-like equilibrium conformation of those particular Calmodulins that were recovered by the corresponding Rd.HMM. Taken toghether these observations reveal a tendency of the Rd.HMM to perform better when built from structures derived from X-ray data.

**Table 4:** Selected hits from the Rd.HMM of the three-dimensional structures for Calmodulin from various sources solved by NMR (PDB entry 1CFF, *Homo sapiens*, 26 structures) or X-ray crystalography (PDB entries 1CLM, from *Paramecium tetraurelia*; 2HQW, from *Rattus norvegicus*; and 1RFJ, from *Solanum tuberosum*).

Starting structure	Hit number	NCBI-nr gi	PDB, SWISS-PROT or RefSeq entries	Score^c^	Log (E-value^c^)	N dom.	Source	Full description
1CFF model 2	1	170594293	XP_001901898.1	19.1	0.041	1	*Brugia malayi*	Calmodulin-like protein
1CFF model 2	6	156083146	XP_001609057.1	15.4	0.462	1	*Babesia bovis* T2Bo	calmodulin
1CFF model 2	65	90109258	2F2O	10.7	0.991	1	*Bos taurus*	Structure Of Calmodulin Bound To A Calcineurin Peptide
1CFF model 8	1	4959599		18.8	0.613	1	synthetic construct	calmodulin mutant SYNCAM60
1CFF model 8	5	122063211	P84339	18.3	0.653	1	*Agaricus bisporus*	CALM_AGABI Calmodulin (CaM)
1CFF model 8	53	4930156	1VRK	15.1	0.959	1	synthetic construct	Structure Of E84k-Calmodulin Rs20 Peptide Complex
1CFF model 18	1	73947271	XP_865498.1	22.1	0.204	1	*Canis familiaris*	PREDICTED: similar to calmodulin 1 isoform 2
1CFF model 18	5	4885111	NP_005176.1	20.3	0.740	1	*Homo sapiens*	calmodulin-like 3
1CFF model 18	6	21465435	1GGZ	20.3	0.740	1	*Homo sapiens*	Structure Of The Calmodulin-Like Protein (Hclp)
1CLM^a^	49	157830637	1CLM	105.1	−24.8	1	*Paramecium Tetraurelia*	Calmodulin at 1.8 Angstroms Resolution
1CLM^b^	3135	33620739	NP_034990.1	17.4	0.996	1	*Mus musculus*	myosin, light polypeptide 6, alkali, smooth muscle and non-muscle
2HQW^a^	4	109502777	XP_001073968.1	122.5	−30.0	1	*Rattus norvegicus*	PREDICTED: similar to calmodulin 1
2HQW^b^	3804	77548632		16	0.991	1	*Oryza sativa* (japonica cultivar-group)	EF hand family protein, expressed
1RFJ^a^	188	55976467	1RFJ	100.4	−23.4	1	*Solanum commersonii*	putative calmodulin
1RFJ^b^	4907	118376630	XP_001021496.1	−22.5	0.996	1	*Tetrahymena thermophila* SB210	EF hand family protein

In the case of NMR models, an individual Rd.HMM (143 ROSETTA-designed sequences) was built for each conformer, only those retrieving at least one sequence from the NCBI-nr sequence database are included. A combined Rd.HMM built with the 2860 sequences from all individual NMR models did not retrieve sequences, and therefore, is not included here. Notes:

a Hit to its own sequence.

b Hit at the bottom of the search with clear DB annotation.

c For the corresponding Rd.HMM.

**Figure 6:**
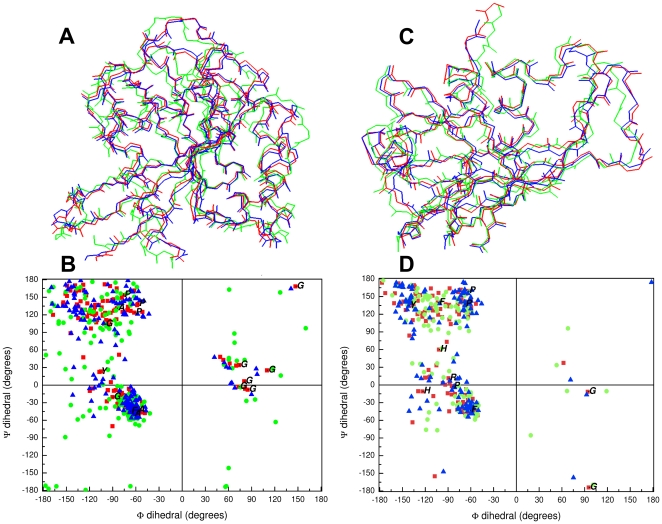
Comparison of the three-dimensional structure of the proteins Ras P21 and RNAse A solved with different experimental methods. A and C) Line-representation of the backbone. Red lines, solved by X-ray diffraction (PDB entries 3X8Y in A, 3LXO in C); green lines, solved by NMR spectroscopy (model 1 only, PDB entries 1CRP in A, 2AAS in C); blue lines, the X-ray data after minimization under Amber94. Relative to the X-ray structure, the backbone RMSD values were 2.32 Å (in A) and 1.1 Å (in C) for the NMR structures, and 0.69 Å (in A) and 0.77 Å (in C), after minimization. B and D) Ramachandran plots of the data in panels A and C, respectively. Red squares, X-ray data (3X8Y in B and 3LXO in D); blue triangles NMR data (1CRP in B and 2AAS in D, only model 1 for both); green circles, X-ray structures after minimization (3X8Y in B and 3LXO in D). Letters indicate the amino acid residues where the Rd.HMM for the corresponding X-ray file was invariant and coincided with the natural amino acid. For the RasP21 protein the Rd.HMM scores against the natural amino acid sequences were 144.3 for 3X8Y, before minimization, 129.7 after minimization, and 11.6 for 1CRP (model 1). For the Rnase A protein the Rd.HMM scores against the natural amino acid sequences were 87.1 for 3XLO, before minimization, 57.4 after minimization, and 42.6 for 2AAS (model 1).

A similar result was obtained when a set of 12 conformers of the Yeast soluble inorganic pyrophosphatase (1E9G) was generated using a 100 ps molecular dynamics simulations at 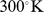 (data not shown). The search retrieved a smaller set of pyrophosphatases from animal and bacterial sources, and the scores were reduced to less than half the score obtained with the Rd.HMM generated for the unmodified PDB file. In addition, two different proteins with known three-dimensional structures, solved by both X-ray crystallography and NMR spectroscopy were analyzed. In both cases, the X-ray structure was also minimized using the Amber-94 forcefield. For the Ras-P21 protein the Rd.HMM for the X-ray structure gave a score 10% higher for the experimental data than after minimization, and the score for the first model in the PDB file of the NMR data was 10 times smaller (Fig. 6). Very similar results were found for the RNAse A, where the Rd.HMM scores were 87.1, 57.4 and 42.6 for the X-ray structure (3LXO), the X-ray structure minimized under Amber 94, and the NMR structure (2AAS, model 1; Fig. 6C), respectively. These observations indicate that a difference must exist between the three-dimensional structures solved by X-ray crystallography, and those solved by NMR. Figure 6 shows the backbone coordinates for the proteins Ras P21 (panel A) and the RNAse A (panel C) solved by X-ray and by NMR, superimposed together along with the X-ray structure after minimization under Amber-94. The overall fold is very similar, but as shown in Figures 6B and 6D, the corresponding Ramachandran plots have important differences. In addition, those invariant positions in the Rd.HMM for the X-ray structure, where the natural sequence was coincident, concentrated on border zones of helical and extended structure, or in the zone of turns (see 1-letter amno acid codes in Figure 6B and 6D). These kind of differences may arise partly from the differences in experimental conditions required for crystallization or for NMR experiments, but also because, in X-ray diffraction data the positions of heavy atoms are known, and in most cases the proton positions are ignored, while NMR renders mainly proton distances, and heavy atom positions must be inferred from there. Since the backbone coordinates are the only information retained during the Rd.HMM protocol, differences in the backbone as those shown in the Figure 6 must be large enough to modify significantly the performance of ROSETTA design and the resulting hidden Markov moldel built by HMMER, and explain the different performance of the X-ray and NMR-solved structures. It may be worth noting that ROSETTA design employs rotamer libraries derived from a set of selected X-ray solved protein three-dimensional structures [24].

As a conclusion from these last data, the Rd.HMM are highly dependent on the backbone coordinates of the starting structure and relatively small deviations in bond lengths, bond angles and dihedrals from those most frequently found in protein crystals (as mentioned before, here, this conformational state is referred to as the crystal-like equilibrium conformation). Deviation from this conformation will result in a reduced score and will increase the probability of a false negative. In contrast, the Rd.HMM searches, given a appropriate cutoff for the E-value (below one) and Rd.HMM score (positive), completely eliminate false positives, as far as we have been able to test them.

### Rd.HMM as tools to test the quality of *in silico* generated 3D-models

Because crystal structures of many different proteins were found to produce essentially the same results described above, we decided to test the ability of 3D-structures of different resolution to retrieve the corresponding amino acid sequence. Ideal data sets for this aim are available from the CASP contests. We used data from the CASP-06 T0315 and the CASP-07 T290 targets, mainly because the crystal structures are already available and because some of the models were below 1.0 Å RMSD from the crystal structure. The results are summarized in Table 5. As expected, the crystal coordinates produced an Rd.HMM with high selectivity, able to retrieve their own sequence from the database with high score and very low E-value. As the 3D-models departed from the crystal structure (increased RMSD, as reported by the CASP staff), the resulting Rd.HMM retrieved its own sequence with smaller score and increasing E-value. The correlation between the RMSD and the Rd.HMM score was low, and this is to be expected, since the Rd.HMM and the RMSD encode the structural information in a very different manner, however, the 3D models considered in Table 5 can be split into three groups: I) those with RMSD below 1 Å found the 2GZX sequence (PDB entry of the X-ray solved three-dimensional structure corresponding to the T0315 CASP-06 target) with high score, though always smaller than the one from the crystal. II) Models with RMSD from 1 to 2 Å were able to recover the target sequence with low scores, but the scores were positive and the E-value was still of statistical significace. III) Models with RMDS above 2 Å recovered a few nonrelated proteins, with low or negative score, or no hits at all.

**Table 5:** Selected hits from the search results for Rd.HMM of the PDB entries 2GZX (one of the targets of CASP 7) and its relative 1J60, and for the Rd.HMM of 3D-models submitted to the CAPS by several contestants.

Contest Position (RMSD rank)^a^	3D-model^b^	Hit number^c^	DB entries:PDB, RefSeq	Description^d^	Score^b^	Log (E-value^b^)	RMSD from the target (Å)
target	PDB entry 2GZX	1	2GZX, YP_001574396.1	TatD family deoxyribonuclease	189.3	−50.09	0
target	PDB entry 2GZX	319	1J6O, NP_228476.1	hypothetical protein	102.6	−24	0
none	PDB entry 1J6O	1	1J6O, NP_228476.1	hypothetical protein	279.7	−77.33	–
none	PDB entry 1J6O	335	2GZX, YP_001245892.1	TatD family hydrolasse	123.1	−30.17	–
1	T0315TS556_1	1	–, YP_002509933.1	hydrolase, TatD family	182.5	−48.07	0.87
1	T0315TS556_1	105	2GZX, YP_185422.1	TatD family deoxyribonuclease	163.9	−42.44	0.87
1	T0315TS556_1	123	1J6O, NP_228476.1	hypothetical protein	160.8	−41.51	0.87
6	T0315TS136_1	1	–, ZP_03557555.1	hydrolase, TatD family	175.8	−46.03	0.88
6	T0315TS136_1	112	2GZX, YP_185422.1	TatD family deoxyribonuclease	151.8	−38.82	0.88
6	T0315TS136_1	163	1J6O, NP_228476.1	hypothetical protein	138.4	−34.8	0.88
12	T0315TS105_1	1	–, YP_002633239.1	putative TatD-related	129.1	−32	0.94
12	T0315TS105_1	14	2GZX, YP_185422.1	TatD family deoxyribonuclease	126.4	−31.18	0.94
12	T0315TS105_1	279	1J6O, NP_228476.1	hypothetical protein	86.4	−19.14	0.94
16	T0315TS675_1	1	–, ZP_01697692.1	hydrolase, TatD family	103.2	−24.19	1.1
16	T0315TS675_1	14	2GZX, YP_185422.1	TatD family deoxyribonuclease	98.3	−22.72	1.1
16	T0315TS675_1	227	1J6O, NP_228476.1	hypothetical protein	67.9	−13.55	1.1
57	T0315TS494_1	1	2GZX, YP_185422.1	TatD family deoxyribonuclease	135.9	−34.03	1.25
57	T0315TS494_1	166	1J6O, NP_228476.1	hypothetical protein	101.4	−23.64	1.25
97	T0315TS186_1	1	–, YP_001907944.1	Putative metal-depen	135.8	−34	1.49
97	T0315TS186_1	354	2GZX, YP_185422.1	TatD family deoxyribonuclease	93.5	−21.27	1.49
97	T0315TS186_1	849	1J6O, NP_228476.1	hypothetical protein	66.4	−13.11	1.49
109	T0315TS383_1	1	–, YP_001882541.1	deoxyribonuclease, TatD family	231.7	−62.89	1.59
109	T0315TS383_1	110	2GZX, YP_185422.1	TatD family deoxyribonuclease	133.8	−33.39	1.59
109	T0315TS383_1	381	1J6O, NP_228476.1	hypothetical protein	126	−31.04	1.59
112	T0315TS474_1	1	1J6O, NP_228476.1	hypothetical protein	106.3	−25.1	1.59
112	T0315TS474_1	425	2GZX, YP_185422.1	TatD family deoxyribonuclease	37	−4.27	1.59
117	T0315TS063_1	1	–, ZP_03224827.1	YabD [Bacillus coahuilensis]	40.8	−5.39	1.76
117	T0315TS063_1	15	2GZX, YP_185422.1	TatD family deoxyribonuclease	37.1	−4.28	1.76
117	T0315TS063_1	855	1J6O, NP_228476.1	hypothetical protein	−23.2	0.11	1.76
120	T0315TS250_1	1	–, YP_415939.1	sec-independent hydrolase	161.5	−41.74	1.97
120	T0315TS250_1	8	2GZX, YP_185422.1	TatD family deoxyribonuclease	160.3	−41.38	1.97
120	T0315TS250_1	338	1J6O, NP_228476.1	hypothetical protein	54	−9.37	1.97
125	T0315AL316_1	1	–, NP_786293.1	metal-dependent hydrolase	86	−19.01	2.71
130	T0315TS511_1	1	–, NP_823412.1	hypothetical protein	−272.8	0.38	3.56

Notes:

a Submitted 3D-models ranked in positions 122 (T0315TS021_1, RMSD 2.24), 123 (T0315TS022_1, RMSD 2.47), 127 (T0315T2193_1, RMSD 2.87) 128 (T0315TS139_1, RMSD 3.03), and 129 (T0315TS054_1, RMSD 3.26) were also analyzed but the searches from the corresponding Rd.HMM rendered empty lists.

b For the corresponding Rd.HMM.

c The hits corresponding to the sequences for the PDB entries 2GZX and 1J60 are shown whenever they appear in the list.

d Only part of the database annotation is shown.

**Figure 7:**
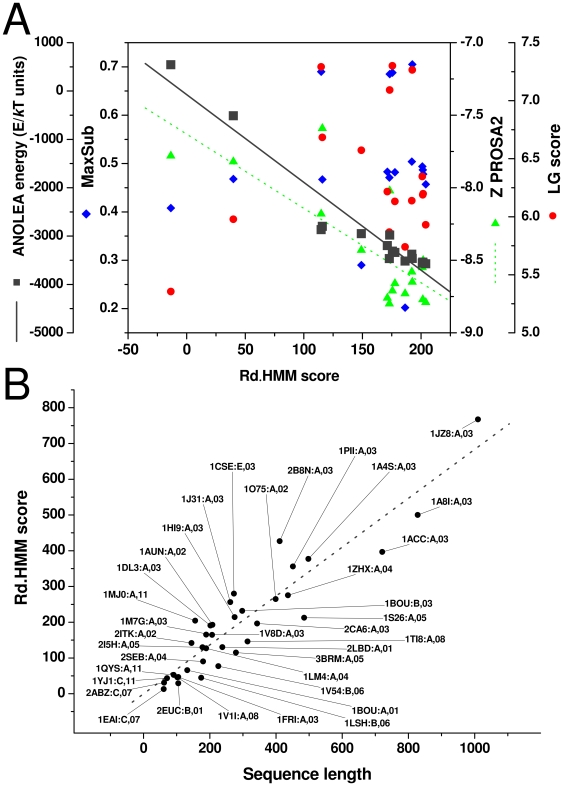
Correlations in the HMM score. A) Rd.HMMscore *vs.* ANOLEA energy and the two ProQ indexes of 17 crystal structures for the Concanavanin A. The structures have been determined to various levels of resolution (given here in parentheses) and were taken from the following PDB entries: 1NLS (0.94Å), 2UU8 (0.94Å), 1JBC (1.15Å), 1QNY (1.80Å), 1DQ6 (1.90Å), 2CTV (1.95Å), 1DQ5 (2.00Å), 2CNA (2.00Å), 5CNA (2.00Å), 1QDC (2.00Å), 1DQ1 (2.15Å), 1ONA (2.35Å), 3CNA (2.40Å), 1QDO (2.80Å), 1DQ4 (2.90Å), 1JOJ (3.00Å), 1XQN (Neutron diffraction). B) Rd.HMM score *vs.* the length of the natural amino acid sequence for a number of X-ray solved three-dimensional structures of proteins from the PDB. Each dot is connected by a line to a label giving the PDB code, the identifier of the chain selected and the SCOP [23] class number of the corresponding structure. At least two representatives of SCOP classes 1 to 8 and 11 are included. SCOP classes 9 and 10 include low resolution data and short peptides; these are not true classes and are not suitable for the Rd.HMM protocol. The Rd.HMM scores given belong to the natural amino acid sequence of the chain corresponding to each structure.

In any case, according to our results, an Rd.HMM from a good 3D-model should be able to retrieve its own sequence, and the higher the Rd.HMM score, the closer the model should be to the experimentally solved 3D structure. To further test this hypothesis, we selected several crystal structures for the same protein. We decided to use the concannavanin A, because there are many files of crystal coordinates in the PDB, the data quality and resolution between them varies significantly, there is even one crystal resolved by neutron diffraction (PDB entry 1XQN), and its structure does not show allosteric transitions or multiple conformations. We built the Rd.HMM for several of them and tried to correlate them against the crystal resolution. The correlation between the resolution of the crystal and the Rd.HMM score was poor (not shown). Again, this is not surprising because there are many factors affecting the quality of X-ray resolved models, such as data set completness, refinement procedures, and the quality of the crystal itself. Instead, we decided to compare this score against well known scores of protein 3D-model quality. Here we included ANOLEA [25], the score used by SWISS-MODEL, PROSA Z-score [26] (https://prosa.services.came.sbg.ac.at/prosa.php), and MaxSub and LGscore from ProQ [27] (http://www.sbc.su.se/~bjornw/ProQ/). The results are shown in Figure 7A, and the best correlation was found for the ANOLEA energy and the Rd.HMM score (r squared 0.931). However, some dispersion occurs at high Rd.HMM scores and low ANOLEA energies. This is the range where the Rd.HMM score becomes rich in information, so Rd.HMM does not replace, but complements the information given by the ANOLEA energy score.

While the Rd.HMM score and E-value give quantitative information of the 3D-model's quality, the strategy reported here renders also a list of the amino acid sequences that a given model represents best, or nothing if the selected database lacks sequences that fit in it. This feature is absent in ANOLEA, PROSA-Z and ProQ, and also in the novel approach implemented in RosettaHoles [28]. RosettaHoles reported a high success in separating PDB entries with acceptable quality from a number of dubious structures [29], but can also separate good from bad protein structural models. Again, as in the case of ANOLEA, the information provided by RosettaHoles is complementary to the one given by the Rd.HMM protocol. Therefore, the ability of Rd.HMM to select amino acid sequences that best fit a given model indicates if the 3D-model under analysis is appropriate, and this constitutes something unique to the Rd.HMM approach.

For Rd.HMM to serve as a good quality-assessment tool, the target score for the model under consideration should be known in advance. Comparison of the data in Figure 3 and Tables 1 and 3 shows important variations in the score of the Rd.HMM for the sequence of its starting protein. As already pointed out, the quality of the crystal does have an effect on the resulting Rd.HMM, but longer proteins appear to score higher. In fact, Figure 7B shows a good linear relationship between the Rd.HMM score and the sequence length. Thus, the line can be used to predict an upper bound for the score. Figure 7B was prepared with an arbitrary sample of 37 different proteins belonging to SCOP [23] classes 1 to 8, and 11. SCOP classes 9 and 10 were not included because they are not true classes and correspond to data that were found to perform poorly in the Rd.HMM protocol, since they contain low resolution data (class 9), and small peptide structures (class 10). A similar plot can be built using the Rd.HMM E-value, however, this plot was parabolic in a log-log scale (not shown), besides, the E-value is dependent on the size of the searched database, with the largest database giving the largest E-value for the same sequence. In contrast, the Rd.HMM score is insensitive to the size of the database searched (not shown). From this plot, the least-squares line has a slope of 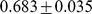 and intercept of 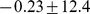 with a r squared of 0.845 (significant with less than 0.0001 error probability). These parameters can be used to predict a target score for a 3D-model, but since an acceptable model is not necessarily identical to a crystal, a simple approximation is to use the lower bound of the straight line, that is, roughly 0.6 times the length of the amino acid sequence under consideration. In addition, the expected Rd.HMM score for the TatD Dnase in Table 5 is known exactly, because the crystal structure is available (2GZX). In this case, proposed 3D models with RMSD values below 1.25 Å from the crystal structure gave an Rd.HMM score of nearly half of the expected value. Therefore, the following rule of thumb can be used a reference: if the 3D-model under consideration shows a Rd.HMM score of 0.3 times its sequence length, then the 3D-model is a good approximation, if it is 0.6 times its sequence length, then it is very close to the crystal-like equilibrium conformation.

**Figure 8:**
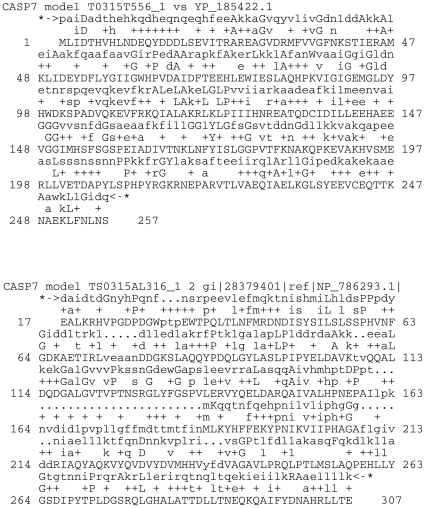
Alignments produced by HMMER search for two different models submitted for the target 315 at the CASP7 contest. The target sequence has RefSeq id YP_185422.1, identical to the 2GZX, except for the N-terminal HIS-tag. Model T0315T556_1 had a score of 163.9 and E-value of 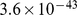 for that sequence. Model TS0315AL316_1 recovered a distantly related sequence of a hydrolase with a TIM-barrel like domain, the Rd.HMM score was 86.0 and the E-value 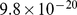. The first line corresponds to the HMMER consensus sequence, the middle line is the HMMER search score mask, and the lowest line is the sequence identified by Rd.HMM in the NCBI-RefSeq database. Dots in the consensus line are gaps.

In addition, Figure 8 shows the alignment report from the Rd.HMM search for the models T0315TS556_1 and T0315AL316_1 corresponding to data in Table 5 with a high score and low score, respectively. As can be seen, the sequence positions (lower line) showing identity or similarity (middle line) to the HMMER consensus (top line) are distributed all along the sequence and there are no gaps (dots in the consensus or dashes in the query sequence). In contrast, the poorly threaded model T0315AL316_1 model retrieved a sequence from a metal-dependent amidohydrolase from *Lactobacillus plantarum* WCFS1, possibly possessing an TIM-barrel like structure (conserved domain number cl00281 in the NCBI-cd database). Yet, the alignment shows less coincidences, and the HMMER algorithm introduced several long gaps to maximize the alignment.

An important feature of the Rd.HMM, deduced from the data in Figure 8, is the relatively low sensitivity of its score to alignment errors between the target and template sequences, frequently introduced during the homology modeling procedure. In the Rd.HMM scheme, ROSETTA design is allowed to generate a completely new amino acid sequence. The information about the original sequence is eliminated and only the information present in the backbone overall geometry is encoded by HMMER. If one selects an appropriate template but produces an improper sequence alignment during the homology-modeling, the final structure may have extended segments in wrong places, but a good part of the 3D-model will still have a low RMSD from the template. Then, during the search, the Rd.HMM scheme will introduce gaps to improve the alignment, thus producing a “corrected” sequence alignment, and bypassing the wrong extensions. While the introduction of gaps penalizes the score, one may still get a meaningful positive score with a badly aligned model, as long as the overall folding is close enough to the correct 3D-structure. In consequence, a good model is characterized by a Rd.HMM score above 0.3 times its sequence length, and by a complete absence of gaps in the HMMER alignment to its own sequence.

It is important to mention that in 8 different examples of the ones included in Figure 7B, the set of ROSETTA-designed sequences generated during the Rd.HMM protocol were analyzed for pseudo-phylogenetic signal, and the results were very similar to the ones obtained with the set from the 1UDE pyrophosphatase (data available upon request). Again, the sequences generated by ROSETTA design appear to be truly independent from each other.

## Discussion

The Rd.HMM scheme is a powerful tool for the assessment of the 3D model of an amino acid sequence, because it gives a quantitative score, and, with positive scores, if the E-value cut-off is set to 1, no false positives are obtained, *i.e.* if the model deviates significantly from the crystal-like equilibrium conformation, its Rd.HMM would not be able to retrieve the amino acid sequence of the modeled protein from a database. Of course, the cost of eliminating the false positives is accepting false negatives, and a Rd.HMM of a 3D-model of a protein may not recover its own sequence and yet be a good starting point, but certainly, it should be still far from an acceptable model.

According to the data presented in this paper, the Rd.HMM of a X-ray solved three-dimensional retrieves from the sequence database only amino acid sequences with the same, or a highly related function to the starting protein; although the amino acids essential to the protein function are not conserved in the sequences generated by ROSETTA design (Fig. 2). At first sight, these two facts may appear as a contradiction, however, it is reasonable to assume that natural selection has finely tuned each three-dimensional structure to meet the functional requirements of every protein, because many functionally important features (such as catalytic and ligand binding sites, or those involved in allosteric trasitions) are known to be extremelly sensitive to even small changes in the local geometry, flexibility, accesible area and other properties. The Rd.HMM encodes structural details by carefully sampling amino acids that can be accomodated into each position of the backbone (Fig. 4B), without seriously increasing the energy, as calculated by Rosetta design. These selections are influenced by the local conformation of the backbone and by the contacts with the residues in the surroundings. In other words, even when many 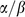 barrels look very similar to each other, each of their corresponding Rd.HMM encodes the structural information with a very fine level of detail, where the presence of secondary-structural elements, their length and the distances between each other are mostly accounted for. While the constraints deriving from functional requirements are absent, the residues essential to the natural function must certainly be amongst the possible Monte Carlo solutions to the structural problem. Then, within the natural sequences, those meeting all these structural requirements, will also meet the functional requirements, and every combination of amino acids providing a functional site matches, in the corresponding Rd.HMM, a set of emitter states able to produce this particular sequence with high probability (amongst many other, without biological meaning). Other 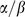 barrels, with overall similarities but different function, will fail to accomodate every single set of functional residues, due to subtle but important differences in the local three-dimensional structure at the positions where these sites should be placed. These considerations should apply to most globular proteins, regardless of their folding pattern.

The Rd.HMM protocol seems to work fine for different types of folds, including membrane proteins, as indicated by the data in Figure 7B, which includes examples from most SCOP classes [23]. It must be said that this protocol has been found to fail in some cases, for instance, the differences in the quality of data in the PDB may vary substantially, and as shown in Figure 7A, data of higher quality, and thus of lower ANOLEA energy, score better in the Rd.HMM protocol, and some may give and empty set in the sequence database search. When there are several structures in the PDB it is often possible to choose the one performing better, but if only one structure is available, structural minimization with molecular mechanics sometimes may improve the quality of the Rd.HMM. Small peptides (less than 100 amino acids long) tend to give low scores, and their corresponding Rd.HMM frequently fail to extract sequences from the databases, and may give a negative value when they are forced to score their corresponding natural sequence. Some unusual proteins may be composed of subunits with a few long elements of secondary structure, which make too few contacts within the subunit (*i.e.* cases where the subunit is a single long 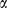-helix, stabilized by subunit-subunit contacts, as in some membrane proteins), while in some other proteins, most of the structure is stabilized by protein-ligand contacts or disulfide bonds. In these cases, the corresponding Rd.HMM search results are frequently empty and the score against their corresponding natural amino acid sequence is negative. Some other causes of failure may be the presence of non-standard, or modified aminoacids (such as selenomethionine, or phophoaminoacids), alternative conformations, records of anisotropic date, missing backbone atoms (including the absence of a C-terminal oxygen), or backbone atoms with zero B-factors. In all of this cases ROSETTA design rejects the coordinates. The easiest way to solve this problems is to delete the conflicting records in the PDB file, change the non-standard aminoacid to their standard counterpart o to reconstruct the missing parts (although this will introduce non-experimental data). What is the best way to proceed depends on the origin of the data, and will affect the quality of the results.

The Rd.HMM can also be used to guide the homology modeling process. The introduction of artificial sequences into the alignments of natural amino acids to aid in the selection of templates for homology modeling has already been demonstrated by Pei et al. [12]. But the Rd.HMM do not seem to need the inclusion of any natural sequence information to be able to detect structural relatives of the starting crystal structure. Thus, if several crystal structures are available for a model, one Rd.HMM can be prepared for each of them, and the best template would be the one recovering the target sequence with the highest score and the smallest E-value. In fact, we have used the alignment of the Rd.HMM search output to guide the alignment of the target sequence and the template sequence in the homology modeling and this strategy seems to have given good results. In addition, if one has doubts about the correct alignment, one can test several alignments, prepare the corresponding models and score all of them with Rd.HMM. While this strategy may by time-consuming, a good and reliable 3D model of a protein is worth the effort. We are now working in the analysis of such schemes and their merits, but this analysis is still in progress, and will be the subject of an upcoming work.

## Materials and Methods

Several 3D structures from the protein data bank (PDB) were selected to contain highly represented folds, rare and synthetic proteins, and representatives of several SCOP [23] classes. This set included the triose phosphate isomerase, the phosphoribosyl anthranylate synthetase, and the 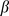-1,4 endoglucanase, as examples of 8-stranded 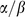 barrels; the TOP7 [10], as an example of a *de novo* designed protein; the putidarredoxin [30], as an example of a rare fold; Calmodulin, a highly mobile protein, and proteins with unknown function (PDB entries 1VK9, 2OEQ, 2NYI, 2P0N, 1MWQ). In addition, we selected the soluble inorganic pyrophosphatases because this group comprises two completely different folds (the Mg-dependent and the Mn-dependent enzymes) and its active form can be obligate hexamers, obligate dimers, or monomers. The ROSETTA program (version 3.3) was run in the *design* mode under the “fixed-backbone” option. The PDB files were used mostly without modification, but amino acids lacking backbone atoms were discarded, because ROSETTA design does not recognize these structures as valid PDB files. Alternative conformations and non-standard amino acids, if present, should also be discarded for the same reason. In order to completely eliminate the original sequence information from the structure, the first set of ROSETTA design runs were performed with a predefined random amino acid sequence indicated in the resource file (ROSETTA resfile). This resulted in 10 or more extremely strained structures, with very high energy scores. Then, each strained structure was “rebuilt” with ROSETTA design but using now a completely unconstrained resource file, *i.e.*, the program was allowed to select any of the 20 amino acids at every position in the structure until energy reached a low value, indicative of a theoretically stable structure. In these runs, ROSETTA energy scores were almost always lower than the energy score of the starting PDB file.

The amino acid sequences were recovered from the resulting sets of ROSETTA-designed PDB files, and aligned. The alignment was trivial because all sequences were of the same length and with perfect biunivocal correspondence to the 3D structure. Alignments done with CLUSTALW [31] reflected this fact so, routinely, the sequences were simply dumped into a fasta file. Only sequences in the rebuilt set were used, neither the native starting sequence, nor the intermediate random sequences were included in the alignment. These alignments were used to generate a hidden Markov model using the HMMER program [15]. The model was calibrated and used to scan the raw NCBI-nr database [32], the NCBI-RefSeq [33], or the NCBI-uniprot_sprot (equivalent to the SWISS-PROT database) for those sequences matching the model. The resulting scoring lists and database-subsets were then analyzed.

In order to produce alignments of ROSETTA-generated sequences for more than one three-dimensional structure, belonging to closely related proteins, structural alignments were produced with TOPOFIT [22], [34], [35]. Because this alignment tool requires substantial computing power, using it for more than 20 or so sequences turned out to be impractical. However, the ROSETTA design program produces structures with an identical backbone, so alignments made with a representative of each set can be simply propagated to the whole set *i.e.*, positions were slid to replicate the gapping pattern. We have prepared some shell scripts (available upon request) to automatically generate the random-sequence intermediates, rebuild them, recover their sequence build the HMMER model and search a local copy of the NCBI-nr, NCBI-RefSeq or NCBI-uniprot_sprot database. Thus the analysis of new protein 3D models can be made with little effort, however the whole process is time-consuming and it may take a day o more, depending on the size of the protein and the power of the computer employed.

A final warning, before starting the procedure the PDB file containing the coordinates to be tested should be made appropriate for ROSETTA design, because some modeling programs may give good folding patterns but with local errors in length, angle or dihedrals of a few bonds, may present missing backbone atoms for one or more residues, or have zero or empty B-factors. ROSETTA design may read these files and produce an output of a small section of the structure, or crash. If this were the case, the PDB file should be edited to contain non-zero B-factors, and if some residues have missing backbone atoms, the full residue may be deleted from the file, or the missing atoms may be reconstructed with a suitable program. Wrong angles and bond-lengths can be corrected with a geometry minimization algorithm under an appropriate force-field.

## Supporting Information

Table S1 Full data from the HMMER search for the Rd.HMM of the soluble inorganic pyrophosphatase from *Saccharomyces cerevisiae* (1E9G), *Pyrococcus horikishii* (1UDE), and from the manganese-dependent enzyme from *Streptococcus gordonii* (1K20) and *Bacillus subtilis* (1WPN, only the N-terminal fragment). The Rd.HMM of the 1WPN structure were built using only the sequences form ROSETTA-design (spreadsheet 5), and these sequences plus the natural amino acid sequence of the protein (spreadsheet 6). The table is a book of Open Office spreadsheets in MS-Excel format. The first spreadsheet contains an index.
(0.61 MB XLS)

Table S2 Full data sets from the HMMER search for the Rd.HMM of several triose phosphate isomerases used to produce tables 1 and 2. This table is a book of Open Office spreadsheets in Excel format. The first spreadsheet contains an index.
(0.48 MB XLS)

Figure S1 Classification of database annotations in the list from Rd.HMM searches corresponding to two enzymes with α/β-barrel three-dimensional structure. The Rd.HMM were generated for the xylanase from *Penicillium simplicissimum* (PDB entry 1BG4; panel A) and the bifunctional enzyme indoleglycerolphosphate synthase/phosphoribosylanthranilate isomerase from *Escherichia coli* (PDB entry 1PII; panel B). The search results lists were classified in bins according to the Rd.HMM score and each bin was subdivided by keywords in a mutually exclusive fashion. In panel A, black bars correspond to sequences annotated as endo-1,4-beta-xylanase, xylanase or Xys1; red bars correspond to those annotated as glycosyl hydrolase, cellobiosidase, or tomatinase and green bars include hypothetical or putative xylanases. In panel B, black bars include sequences annotated as bifunctional or fused Indol-3-glycerol-phosphate synthase/phosphorybosyl anthanilate isomerase; res bars include sequences annotated only as phosphorybosyl anthanilate isomerase and green bars include those annotated only as Indol-3-glycerol-phosphate synthase. In all three panels, cyan bars correspond to predicted, hypothetical or putative proteins, and magenta bars include everything else.
(0.19 MB TIF)

Figure S2 Structurally aware alignment of K^+^ channels obtained from the Rd.HMM built with the KCSA K^+^ Channel (PDB 3F5W). The Rd.HMM was prepared as described in the Materials and Methods section and the individual alignments in the HMMER search results were merged using the HMMER consensus (first sequence in the alignment) as a guide (see also Figure 8). The figure was prepared using JalView [Waterhouse *et al.* (2009) *Bioinformatics* 25: 1189–91].
(1.99 MB TIF)
