# Supplementary figures and images for: A Score of the Ability of a Three-Dimensional Protein Model to Retrieve Its Own Sequence as a Quantitative Measure of Its Quality and Appropriateness
Source: PLoS One. 2010 Sep 7;5(9):e12483. doi: 10.1371/journal.pone.0012483 (PMC2935356; doi:10.1371/journal.pone.0012483)

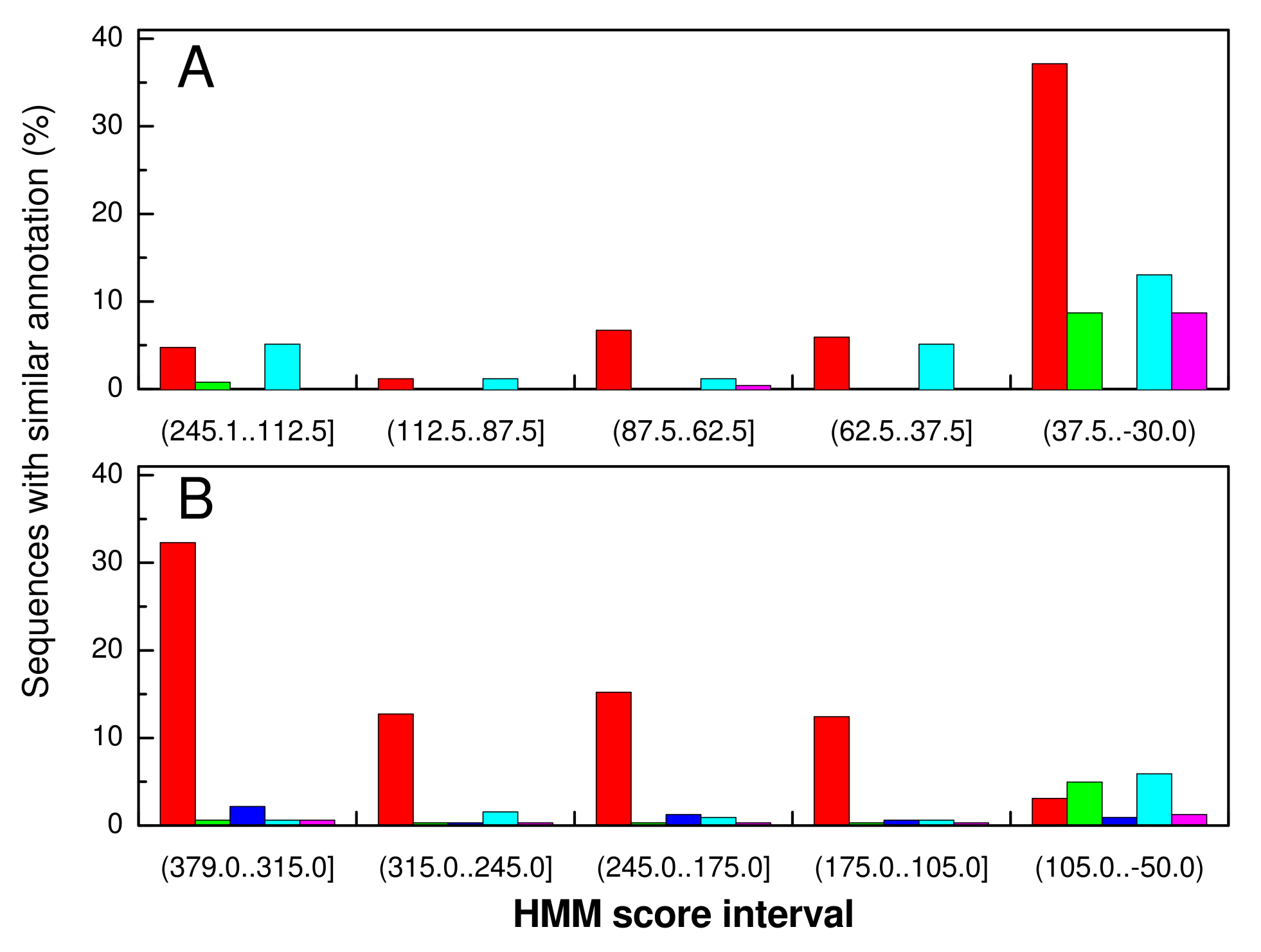

Supplement: Figure S1 — Classification of database annotations in the list from Rd.HMM searches corresponding to two enzymes with α/β-barrel three-dimensional structure. The Rd.HMM were generated for the xylanase from Penicillium simplicissimum (PDB entry 1BG4; panel A) and the bifunctional enzyme indoleglycerolphosphate synthase/phosphoribosylanthranilate isomerase from Escherichia coli (PDB entry 1PII; panel B). The search results lists were classified in bins according to the Rd.HMM score and each bin was subdivided by keywords in a mutually exclusive fashion. In panel A, black bars correspond to sequences annotated as endo-1,4-beta-xylanase, xylanase or Xys1; red bars correspond to those annotated as glycosyl hydrolase, cellobiosidase, or tomatinase and green bars include hypothetical or putative xylanases. In panel B, black bars include sequences annotated as bifunctional or fused Indol-3-glycerol-phosphate synthase/phosphorybosyl anthanilate isomerase; res bars include sequences annotated only as phosphorybosyl anthanilate isomerase and green bars include those annotated only as Indol-3-glycerol-phosphate synthase. In all three panels, cyan bars correspond to predicted, hypothetical or putative proteins, and magenta bars include everything else. (0.19 MB TIF) [file pone.0012483.s003.tif]

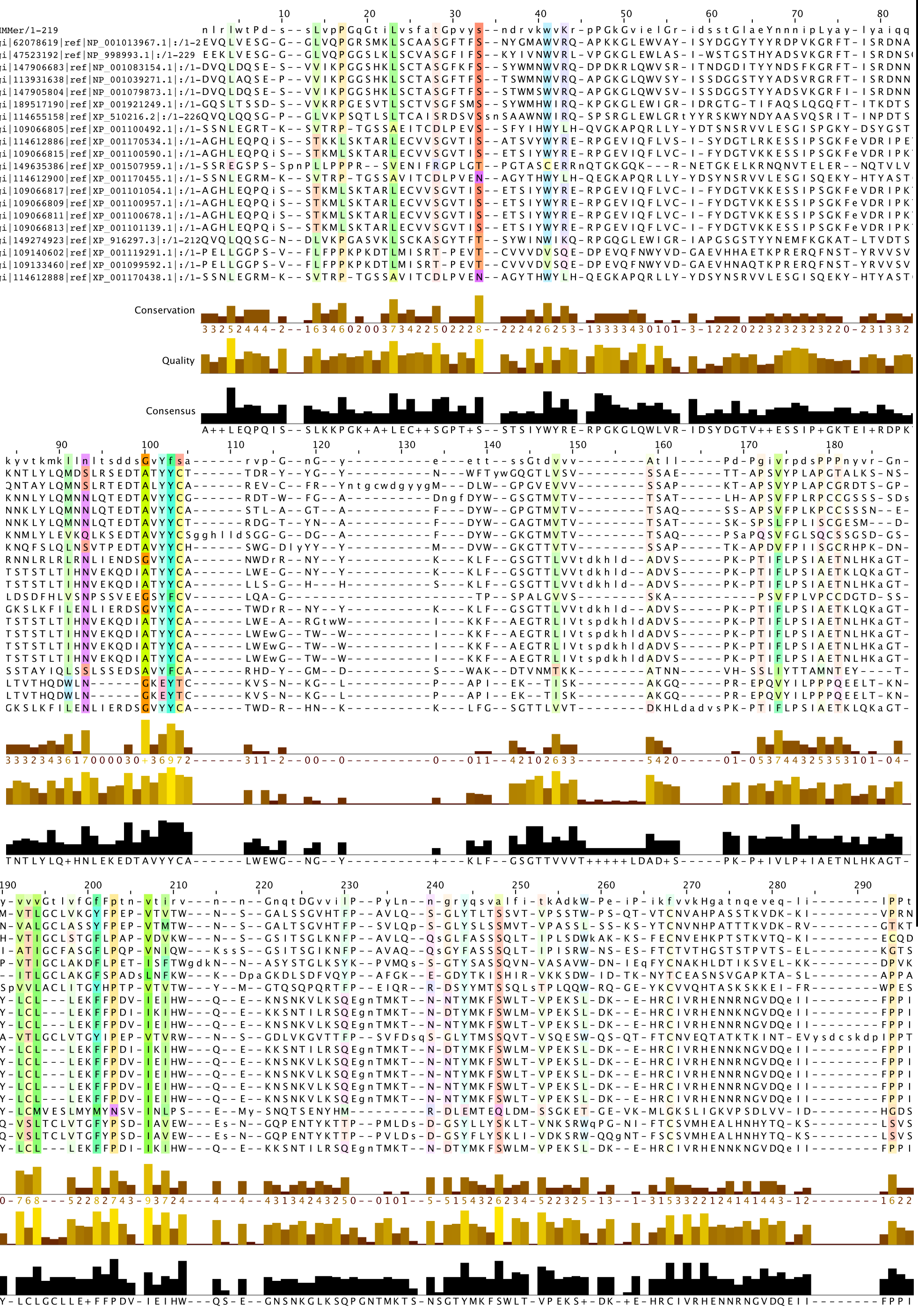

Supplement: Figure S2 — Structurally aware alignment of K+ channels obtained from the Rd.HMM built with the KCSA K+ Channel (PDB 3F5W). The Rd.HMM was prepared as described in the Materials and Methods section and the individual alignments in the HMMER search results were merged using the HMMER consensus (first sequence in the alignment) as a guide (see also Figure 8). The figure was prepared using JalView [Waterhouse et al. (2009) Bioinformatics 25: 1189–91]. (1.99 MB TIF) [file pone.0012483.s004.tif]
